# The Role of Laparoscopic Nephrectomy in Pediatric Xanthogranulomatous Pyelonephritis: A Case Report

**DOI:** 10.1155/2013/598950

**Published:** 2013-02-27

**Authors:** James F. Brown, Jennifer C. Chamberlain, Christopher C. Roth

**Affiliations:** Department of Urology, Louisiana State University Health Sciences Center and Children's Hospital of New Orleans, 200 Henry Clay Avenue, New Orleans, LA 70118, USA

## Abstract

Xanthogranulomatous pyelonephritis (XGP) is a rare chronic renal infection characterized by the destruction of renal parenchyma. Traditional treatment involves open radical nephrectomy, which is challenging due to the inflammatory process associated with XGP. More recently, laparoscopic nephrectomy has been employed successfully in adult XGP. We present a case of a six-year-old female child with XGP who was successfully treated by laparoscopic nephrectomy with minor complications. Our case demonstrates the safety and feasibility of laparoscopic nephrectomy for childhood XGP, indicating that it should be considered a management option in such cases.

## 1. Introduction

Xanthogranulomatous pyelonephritis (XGP) is a rare chronic renal infection characterized by destruction of renal parenchyma and replacement with sheets of foamy macrophages [1]. The disease is commonly associated with nephrolithiasis and upper tract obstruction and more commonly affects middle-aged women [2].

Traditional treatment involves radical nephrectomy because symptomatology and preoperative imaging cannot reliably distinguish XGP from neoplastic processes. Nephrectomy is challenging because of the dense inflammatory process surrounding the kidney [3]. For most benign conditions where nephrectomy is indicated, laparoscopy has become the modality of choice due to shorter hospital stays, decreased postoperative pain, and timely return to full activity. 

Historically, laparoscopic nephrectomy (LN) was contraindicated or had shown no benefit compared to open nephrectomy for XGP [4–6]. However, more recent studies have shown that in experienced hands, LN can be performed with excellent results. There are few reports on LN for XGP in children, one which focuses solely on a retroperitoneal approach [7]. We report on a female pediatric patient with congenital obstruction who successfully underwent transperitoneal laparoscopic nephrectomy for XGP.

## 2. Case Report

A six-year-old female child presented with a three-week history of mild left flank pain and low-grade fever. Her medical history was unremarkable, with no history of previous urinary tract infections. A routine urinalysis was suggestive of UTI. She was transferred to our care after her symptoms failed to resolve with oral antibiotics.

**Figure 1:**
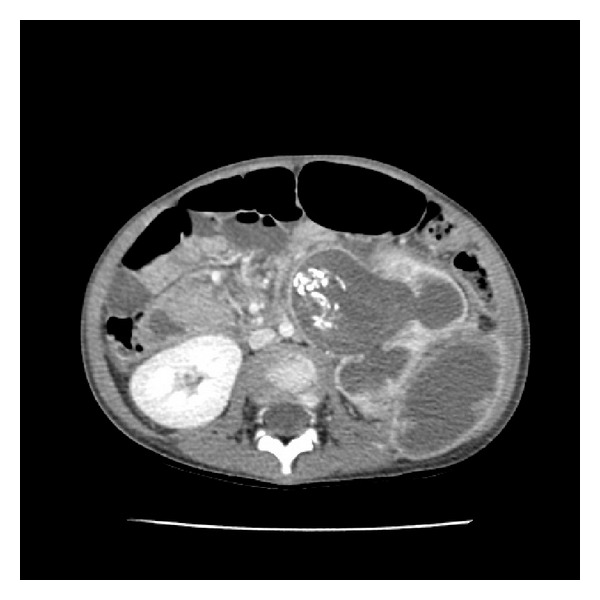
Predrainage CT with contrast demonstrates calcified debris filling the pelvis and proximal ureter. There is destruction of the lower pole renal parenchyma (not pictured) which is contiguous with a 7 × 6 × 5 cm abscess and destruction of the upper pole renal parenchyma with a 4 × 3 × 2 cm abscess.

Physical exam at presentation demonstrated a temperature of 38.3°C and a palpable left flank mass with overlying fluctuance. Laboratory evaluation demonstrated a hemoglobin of 8.5 g/dL and a white blood cell count of 18.6 × 10^3^/*μ*L with 84% segmented neutrophils. A voided urine culture was obtained which was later positive for *E. coli.* Abdominal CT demonstrated a chronically hydronephrotic left kidney with calcified debris within the collecting system and perinephric abscesses extending anterior and posterolateral to the kidney (Figure 1). 

The patient was admitted for IV antibiotics, percutaneous perirenal abscess drainage, and nephrostomy. 120 cc and 30 cc of purulent material were drained from the abscess and nephrostomy, respectively. Initial and subsequent cultures demonstrated *E.coli.* The patient responded well to drainage and was treated with an extended course of culture-specific antibiotics. A dimercaptosuccinic acid renal scan after one month of percutaneous drainage revealed that the left kidney had a differential function of 5%. 

**Figure 2:**
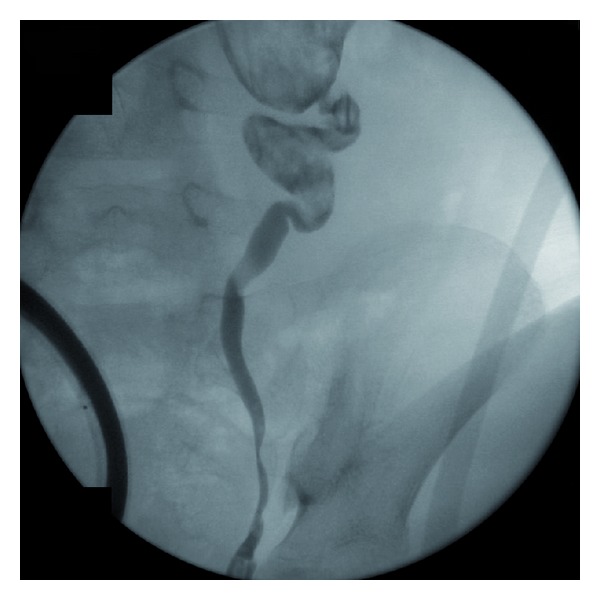
A retrograde pyelogram prior to surgery shows opacification of the dilated left renal collecting system with abundant internal debris noted within the renal pelvis and proximal ureter. The ureteropelvic junction appears narrowed, though obstruction elsewhere along the ureter and dilating reflux cannot be ruled out.

A left retrograde pyelogram was performed at the time of definitive surgery in order to identify the distal extent of the calcified debris and to demonstrate areas of potential obstruction. Fluoroscopy demonstrated a narrowing at the ureteropelvic junction with migration of calcified debris into the proximal ureter (Figure 2). These findings do not exclude vesicoureteral reflux as a potential cause of her chronic pyelonephritis.

We elected for a transperitoneal left LN secondary to broader visualization, superior angles, and similar outcomes data compared to a retroperitoneal approach [8]. Trocar placement was as follows. A 10 mm camera trocar was placed in the umbilicus. Two working trocars were placed in the left midclavicular line: an infraumbilical 12 mm trocar and a supraumbilical 5 mm trocar. An assistant 5 mm trocar was placed in the midline below the xiphoid process. A Harmonic Scalpel was the primary energy source for dissection. Retroperitoneal access was achieved distally near the iliac vessels by reflecting the colon medially. The retroperitoneum was expectantly inflamed, requiring methodical dissection. Inspection of the kidney revealed dense perinephric adhesions with prominent inflammation near the upper pole. The renal vessels were ligated en bloc with an endoscopic vascular stapler. An inadvertent capsulotomy was created at the upper pole resulting in leakage of inflammatory debris. A Covidien *Endocatch* bag was used to deliver the specimen through an enlarged incision at the site of the 12 mm trocar. The abdomen was irrigated with one liter of bacitracin, and the irrigant and debris were aspirated with an endoscopic sucker. The total operative time was 286 minutes. Estimated blood loss was 100 cc. 

The postoperative course was complicated by an ileus (Clavien grade II) requiring nasogastric tube decompression. This resolved by postoperative day three and the patient was discharged home on postoperative day five. 

**Figure 3:**
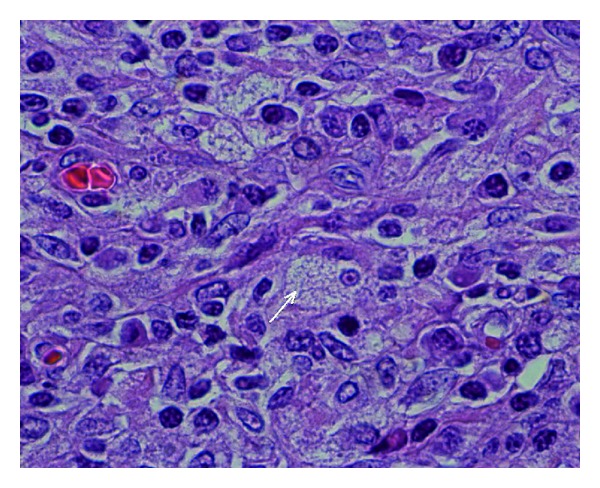
Numerous foamy histiocytes forming granulomas with occasional plasma cells and lymphocytes (1000x image).

Histology demonstrated marked chronic interstitial nephritis, tubular atrophy, fibrosis, and pelvic and muscular wall hypertrophy. Collections of foamy histiocytes admixed with lymphocytes and plasma cells were noted under the urothelium (Figure 3). 

## 3. Discussion

The first case of pediatric XGP was described in 1963 and approximately 265 subsequent cases have been reported in the literature [9, 10]. The disease is commonly associated with obstructive uropathy by nephrolithiasis or, as in our patient, congenital obstruction [11]. Some reports have also associated XGP with renal ischemia, lymphatic obstruction, immunodeficiency, and bacteremic seeding [9, 12]. The most common causative organisms are *E. coli* and Proteus [12]. Nephrectomy is curative in unilateral disease. Until recently, open radical nephrectomy was preferred over laparoscopy for XGP. Due to improving surgical technique and reports of good outcomes in recent years, LN has reemerged as a procedure of choice. 

Minimally invasive surgery for renal disease has become commonplace since the first laparoscopic nephrectomy in 1991; however, early experience contraindicated its use for XGP [6]. Because it frequently involves the renal pelvis, hilum, and adjacent structures, XGP presents significant challenges to surgeons [6]. Procedural difficulty and lack of evidence for decreased morbidity led to early recommendations against LN, notably by Bercowsky et al. [4]. In 2005, Khaira et al. [11] concluded that complication rates between LN and open nephrectomy are equivalent, but noted lower analgesic requirements and shorter hospital stays. These findings are further supported by other recent case series [5, 6, 13]. Consequently, surgeons now consider minimally invasive approaches to XGP. Provided the ideal clinical scenario, a low threshold for open conversion, and an experienced surgeon, the benefits of laparoscopic surgery can extend to XGP patients [6]. 

Currently, most case series addressing the role of laparoscopy in XGP are limited to adult patients [7]. A study from Birmingham Children's Hospital in the UK [7] provides the only current case series of LN for pediatric XGP. This study describes three children who underwent retroperitoneal LN with minimal complications, none requiring transfusion or open conversion. Postoperative wound infection (grade II), pyrexia (grade I), and ileus (grade II) were reported among the three patients. These complications resolved promptly, with a maximum hospital stay of seven days. The Birmingham authors acknowledge the challenges of laparoscopy for XGP, but recommend it as the procedure of choice [7]. 

Our paper addresses the role of laparoscopy for pediatric XGP, a subject represented by few reports in the literature. As expected, the operation presented challenges, including purulent spillage and the need for careful dissection from adjacent structures. These procedural difficulties were addressed and overcome. Our patient required a short postoperative stay, with timely resolution of her ileus. By demonstrating a good outcome following prudent use of laparoscopic nephrectomy, our paper corroborates the findings of other contemporary case reports and broadens their application to pediatric surgery. We recommend considering LN for pediatric cases. Under the hands of experienced surgeons, the therapeutic and cosmetic benefits of LN can serve XGP patients. 

Our case demonstrates successful treatment of pediatric XGP with LN, strengthening the role of laparoscopy as a treatment for this disease. Though initial studies recommended against its use for XGP, LN is now reemerging as a procedure of choice thanks to successes documented in recent adult case reports [6]. Our case extends these favorable outcomes to pediatric surgery. Acknowledging its challenges and limitations, skilled surgeons should seriously consider LN for the treatment of childhood XGP. 

## Abbreviations

XGP: Xanthogranulomatous pyelonephritis
LN: Laparoscopic nephrectomy
UTI: Urinary tract infection
CT: Computed tomography.
